# Amino acids in piglet diarrhea: Effects, mechanisms and insights

**DOI:** 10.1016/j.aninu.2023.07.009

**Published:** 2023-10-24

**Authors:** Xihong Zhou, Jing Liang, Xia Xiong, Yulong Yin

**Affiliations:** a Key Laboratory of Agro-ecological Processes in Subtropical Region, Institute of Subtropical Agriculture, The Chinese Academy of Sciences, Changsha 410125, China; b College of Advanced Agricultural Sciences, University of Chinese Academy of Sciences, Beijing 100049, China

**Keywords:** Piglet, Amino acid, Diarrhea, Gut microbe, Immune

## Abstract

Piglet diarrhea is among one of the most serious health problems faced by the pig industry, resulting in significant economic losses. Diarrheal disease in piglets has a multifactorial etiology that is affected by physiology, environment, and management strategy. Diarrhea is the most apparent symptom of intestinal dysfunction. As a key class of essential nutrients in the piglet diet, amino acids confer a variety of beneficial effects on piglets in addition to being used as a substrate for protein synthesis, including maintaining appropriate intestinal integrity, permeability and epithelial renewal, and alleviating morphological damage and inflammatory and oxidative stress. Thus, provision of appropriate levels of amino acids could alleviate piglet diarrhea. Most amino acid effects are mediated by metabolites, gut microbes, and related signaling pathways. In this review, we summarize the current understanding of dietary amino acid effects on gut health and diarrhea incidence in piglets, and reveal the mechanisms involved. We also provide ideas for using amino acid blends and emphasize the importance of amino acid balance in the diet to prevent diarrhea in piglets.

## 1 Introduction

Diarrhea is among one of the most serious piglet health problems, resulting in significant economic losses in the pig industry. Diarrheal disease in weaned piglets has a multifactorial etiology that is affected by physiology, environment, and management strategies. These factors are influenced by interactions between pathogens, host immunity, diet, and farm procedures. As the most apparent indicator of intestinal dysfunction, diarrhea commonly reflects the inability of the intestine to maintain water and electrolyte homeostasis. Weaning stress causes malabsorption of nutrients and decreases the net absorption of electrolytes and fluids by the intestine, leading to the development of piglet diarrhea (Heo et al., 2013).

Host regulation of intestinal mucosal barrier permeability is critical for nutrient uptake and defense against invasion by pathogens and harmful substances. Factors that increase intestinal permeability can induce diarrhea via the pore and leak pathways by increasing tight junction permeability or via unrestricted pathways by causing epithelial damage (Tsai et al., 2017). The most prevalent causes of diarrheal diseases in piglets are pathogens, including the bacterial species *Escherichia coli* and *Salmonella* spp., viruses such as porcine epidemic diarrhea virus, nematodes, and protozoan parasites. Nutritional status is also a major cause of diarrhea that can affect diarrhea-related morbidity and mortality. Freshly weaned piglets undergo a transition from easily digestible liquid milk to more complex, less digestible solid feed, altering intestinal morphology and resulting in inflammatory responses, thereby inducing diarrhea. High dietary protein levels in post-weaned piglet diets induce a higher incidence of diarrhea, since high protein can stimulate allergic reactions and intestinal dysbiosis, and undigested proteins can be converted to toxic substances in the hindgut (Xia et al., 2022; Yin et al., 2021). Thus, managing dietary protein levels and composition can effectively treat diarrhea and reduce fecal output (de Mattos et al., 2009). Early weaning is an important cause of diarrhea in piglets via over-accumulation of reactive oxygen species (ROS) resulting in significant alterations in the digestive tract, including villus atrophy, crypt hyperplasia, elevated pH, and reduced digestive enzyme activity (Xia et al., 2022). These changes enhance susceptibility to pathogen invasion and diarrhea.

**Fig. 1:**
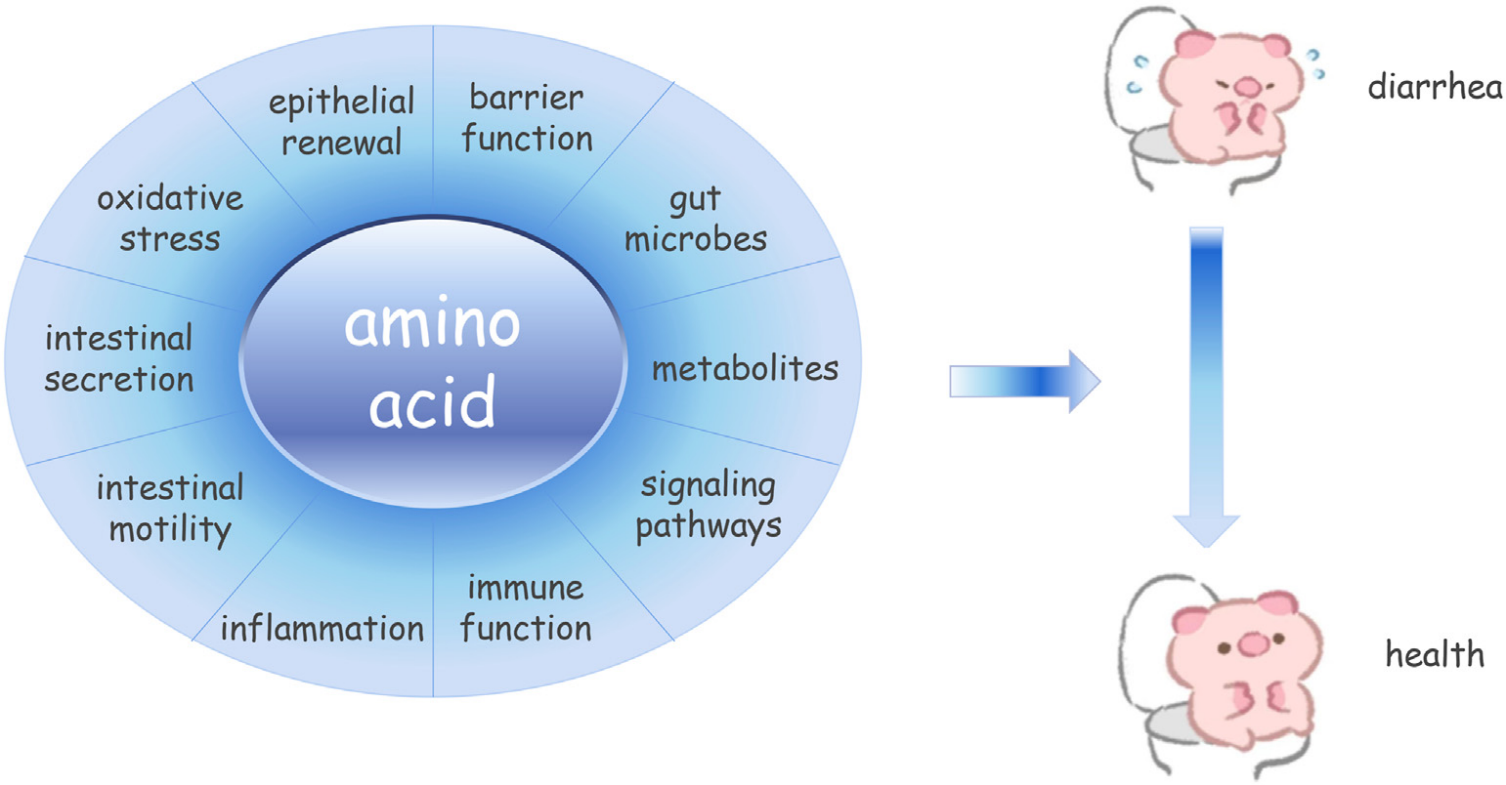
An overview of amino acid mediated mechanisms impacting piglet diarrhea. As a key class of essential nutrients in the piglet diet, amino acids confer a variety of beneficial effects in piglets in addition to being used as a substrate for protein synthesis, including balancing intestinal secretion and absorption, maintaining appropriate intestinal motility, integrity, permeability and epithelial renewal, and alleviating morphological damage and inflammatory and oxidative stress. Thus, provision of appropriate levels of amino acids could alleviate piglet diarrhea. Most amino acid effects are mediated by metabolites, gut microbes, and related signaling pathways.

As a key class of essential nutrients in the piglet diet, amino acids confer a variety of beneficial effects on piglets in addition to being used as a substrate for protein synthesis, including maintaining appropriate intestinal integrity, permeability and epithelial renewal, and alleviating morphological damage and inflammatory and oxidative stress and improving microbiota composition (Fig. 1). Thus, appropriate levels of amino acids are commonly supplemented in weaned piglets for maintaining gut health and preventing diarrhea. In this review, we summarize the current understanding of dietary amino acid effects on gut health and diarrhea incidence in piglets, and reveal the mechanisms involved. We also provide ideas for using amino acid blends and emphasize the importance of amino acid balance in the diet to prevent diarrhea in piglets.

## 2 An overview of amino acid mediated mechanisms impacting piglet diarrhea

### 2.1 Amino acid effects on intestinal development and function

Amino acids are essential for the formation of intestinal structures and maturation of function (Table 1), which is often impaired in early weaned piglets. Intestinal developmental disorders and dysfunction result in low digestive, absorptive, and mucosal barrier abilities and weak immune capacities, elevating the incidence of post-weaning diarrhea syndrome and death in piglets (Cheng et al., 2018). Amino acids can be used catabolically for energy, and anabolically for protein synthesis. They are thus closely involved in promoting intestinal tract development and repair of mucosal barrier injury by improving epithelial cell proliferation and differentiation (Xia et al., 2022). Importantly, functional amino acids are critically involved in regulating gene expression, post-translational modifications, and signal transduction, and further affect inflammatory and oxidative responses and immune function (Mou et al., 2019). Amino acids target signaling pathways, including angiotensin-converting enzyme 2 (ACE2), nuclear factor erythroid-related factor 2 (Nrf2), mitogen-activated protein kinase (MAPK), inducible nitric oxide synthase (iNOS), mammalian target of rapamycin (mTOR), calcium-sensing receptor (CaSR), general controlled non-repressed kinase 2 (GCN2), and nuclear factor-kappa B (NF-κB) (He et al., 2018). Additionally, several studies have suggested that an amino acid-based diet may alleviate diarrhea by increasing water intake (Kimizuka et al., 2021) and eliminating intestinal inflammation (He et al., 2018; Liu et al., 2017).

**Table 1:** Effects of amino acids on growth performance, diarrhea occurrence and intestinal function in piglets.

AA	Experiment period	Dosage	Growth performance	Diarrhea	Intestinal function	References
Gln	Pre- and post-weaning piglets	1%	Increased ADG, ADFI and feed conversion	Lower diarrhea ratio and shorter diarrhea duration	Promote NaCl absorption; increase absorption capacity for xylose and mannitol; improve oxidative stress and immune function	Rhoads et al. (1990); Wang et al. (2015); Zou et al. (2006)
BCAA	Weaning piglets for 14 d	0.19% Ile, 0.27% Val, 0.07% Leu	Increased ADG, ADFI and feed conversion	Not observed	Increase villus height and immunoglobulin level; maintain intestinal barrier function and enhance enterocyte proliferation	Ren et al. (2015)
Leu	From birth to 21 d old	0.95 kg/kg BW	Increased ADG	Not observed	Improve intestinal development; reduce ROS level	Hu et al. (2017); Sun et al. (2105)
Ile	Weaning piglets for 17 d	0.4%	Increased ADG and ADFI	Not observed	Improve intestinal development; alter gut microbiota composition	Goodarzi et al. (2022); Ren et al. (2019); Zhang et al. (2016)
Trp	Weaning piglets for 4 weeks	From 0.2% to 0.4%	Increased ADG and ADFI	Decrease diarrhea rate and index	Alter gut microbiota composition; enhance barrier function; alleviate oxidative stress, inflammation and apoptosis	Kim et al. (2010); Liang et al. (2018a, b); Liu et al. (2019); Rao et al. (2021)
Arg	Weaning piglets for 1 week	From 0.4% to 1.6%	Increased ADG and feed conversion	Decrease diarrhea incidence at low level and increase diarrhea incidence at high level (1.6%)	Suppress inflammatory cytokine expression; improve intestinal and microvascular development; enhance immune status; alleviate oxidative stress	Shan et al. (2012); Yao et al. (2011); Zhan et al. (2008); Zheng et al. (2017)
Met	Weaning piglets for 2 weeks	From 0.15% to 0.35%	Increased ADG and feed conversion	Not observed	Maintain the integrity and barrier function; alter gut microbiota composition	Chen et al. (2014); Kaewtapee et al. (2016)
Cys	Weaning piglets for 3 weeks	From 0.25% to 0.5%	Not affected	Attenuate diarrhea in mice; not observed in piglets	Improve intestinal mucosal integrity and epithelial cell turnover; attenuate intestinal inflammation and oxidative stress	Song et al. (2016); Yoneda et al. (2021)
Ser	Weaning piglets for 4 weeks	0.2%	Increased ADG	Decrease diarrhea incidence	Improve intestinal integrity, inflammation and oxidative status	Zhou et al. (2018a, b)
Gly	Weaning piglets for 4 weeks	From 1% to 2%	Increased ADG and feed conversion	Not observed	Improve intestinal mucosal morphology, antioxidant capacity and apoptosis; regulate mucosal immunity and microbial composition; improve energy status and protein synthesis	Ji et al. (2022); Xu et al. (2018); Yang et al. (2022)
Lys	Weaning piglets for 3 weeks	Deficiency	Increased feed intake	Not observed	Alter gut microbiota composition	Yin et al. (2017a, b, 2018)
Thr	Weaned piglets for 2 weeks; 2-d-old piglets for 8 d	Deficiency	Not affected	Increase diarrhea score	Affect paracellular permeability and glucose absorption capacity; villus hypotrophy; lower mucosal mass and total crude mucin content	Hamard et al. (2010); Law et al. (2007)

AA = amino acid; Gln = glutamine; BCAA = branched-chain amino acids; Val = valine; Leu = leucine; Ile = isoleucine; Trp = tryptophan; Arg = arginine; Met = methionine; Cys = cysteine; Ser = serine; Gly = glycine; Lys = lysine; Thr = threonine; ADG = average daily weight gain; ADFI = average daily feed intake; ROS = reactive oxygen species.

### 2.2 Gut microbes mediate amino acid effects on piglet diarrhea

The intestinal microbiota plays a critical role in maintaining intestinal health in weaned piglets. Various anaerobic bacteria adhere to the mucosa surface and interact with the intestinal epithelium (Kumar et al., 2014). These bacteria form a barrier that can resist exogenous pathogen invasion, and disruption of this barrier causes intestinal injury and induces piglet diarrhea. Diarrhea is often associated with dysbiosis of the gut microbiota, and piglets with and without diarrhea show different microbiota characteristics. Piglets predisposed to diarrhea are usually characterized by an increased abundance of Actinobacteria and *Prevotella,* and a decreased abundance of *Lactobacillus* and *Bacteroides*, whereas piglets with high resistance to diarrhea during weaning display higher abundance of *Chlamydia* or *Helicobacter* in their feces (Gryaznova et al., 2022; Karasova et al., 2021; Ren et al., 2022; Zhou et al., 2022). Various studies have reported that amino acids, including methionine, tryptophan, glycine, threonine, and lysine, affect the growth and composition of microbes in the intestinal lumen. The specific effects of individual amino acids on gut microbes are discussed below.

### 2.3 Amino acid metabolite effects on piglet diarrhea

Amino acid effects on diarrhea can be attributed to their direct effects on the intestine or the effects of their metabolites, including nitric oxide (NO) produced in the small intestine, and 5-hydroxytryptophan (5-HT) produced in the hindgut. Additionally, fermentation pathways of gut microbiota in the large intestine can produce a variety of substances from excess proteins, including indole, spermidine, putrescine, hydrogen sulfide (H_2_S), ammonia nitrogen, and histamine, generated by amino acid deamination (Windey et al., 2012). Although some metabolites, such as 5-HT and melatonin, are beneficial for gut health, over-accumulation of many metabolites impairs barrier structure and function, increases mucus barrier permeability, and causes inflammatory responses and diarrhea in weaned piglets (Rist et al., 2013).

## 3 Effects of specific amino acids on piglet diarrhea and their mechanisms of action

### 3.1 Glutamine

Glutamine is the primary fuel for enterocytes, and the small intestine utilizes approximately two-thirds of the dietary glutamine ingested by piglets. Diets supplemented with glutamine provide substrates for the synthesis of nucleotides, which are indispensable for proliferating cells, and upregulate expression of genes involved in cell growth and renewal (Wang et al., 2008), thus exerting beneficial effects countering intestinal epithelial cell impairment in weaned piglets (Liao, 2021). Glutamine exerts its effects on intestinal oxidative stress via providing a substrate for glutathione synthesis (Wang et al., 2008), and on inflammatory responses via regulating signaling pathways including mTOR, MAPK, adenosine 5’-monophosphate-activated protein kinase (AMPK) and NF-κB (He et al., 2022; Sakiyama et al., 2009; Zhu et al., 2015). Glutamine improves intestinal permeability and electroneutral sodium chloride absorption, and minimizes downregulation of tight junction protein expression in the small intestine of piglets under weaning stress (Ewaschuk et al., 2011; Rhoads et al., 1990; Wang et al., 2015). Diets enriched with glutamine protect the host from bacterial invasion by inhibiting bacterial adherence to intestinal epithelial cells (Souba et al., 1990). These effects of glutamine indicate its potential for preventing diarrhea. Evidence suggests that a diet supplemented with 1.0% glutamine reduces diarrhea prevalence and duration in weaned piglets (Zou et al., 2006).

### 3.2 Branched-chain amino acids (BCAA)

In addition to glutamine and glutamate, BCAA are considered an energy source for rapidly proliferating enterocytes and nutrient transport. This could explain why BCAA play important roles in maintaining intestinal barrier function and improving morphology by increasing villus height in the small intestine (Ren et al., 2015; Sun et al., 2015). In addition to their role as a nutrient substrate, BCAA could serve as signal molecules in pathways that regulate protein turnover, lipid and glucose metabolism, redox balance, and immune defenses primarily via targeting mTOR and NF-κB (Hu et al., 2017; Marc Rhoads and Wu, 2009; Ren et al., 2019). Notably, it has been suggested that dietary isoleucine can upregulate the expression of Na^+^/glucose co-transporter 1 (SGLT1) in the small intestine (Zhang et al., 2016), indicating its potential role in Na^+^ absorption. Unfortunately, to our knowledge, there is no direct evidence showing the effects of BCAA on diarrhea in weaned piglets. Additionally, although several studies have suggested that BCAA could be used by bacteria and might beneficially affect intestinal microbiota composition (Goodarzi et al., 2022; Zhou et al., 2018a), results regarding their influence on gut microbes in weaned piglets are still scarce, and whether these alterations are involved in alleviating diarrhea needs to be further elucidated.

### 3.3 Aromatic amino acids (AAA)

Weaning stress in piglets is characterized by intestinal inflammation, and increased dietary levels of AAA are required to prevent an inflammatory immune response (Gao et al., 2018). Diets supplemented with a combination of AAA have been reported to improve amino acid utilization and alleviate inflammation by enhancing CaSR levels in weaned piglets (Duanmu et al., 2021; Liu et al., 2018). Tryptophan, a nutritionally essential amino acid, is widely used in piglet diets and exerts various beneficial effects on the intestine, including the alleviation of inflammation, endoplasmic reticulum stress, and apoptosis, as well as improving morphology and integrity in different models of weaned piglets, including those challenged with lipopolysaccharide, diquat, or dextran sodium sulfate (Kim et al., 2010; Liang et al., 2018a; Liu et al., 2019, 2022; Rao et al., 2021). Thus, dietary tryptophan reduces the diarrhea rate and index in weaned piglets (Rao et al., 2021). Additionally, combined supplementation with tryptophan and threonine shortened the time for which antibiotic use was required to prevent diarrhea in weaned piglets (Engelsmann et al., 2023). Indirect evidence has shown the involvement of tryptophan metabolism in the occurrence of diarrhea. For example, *Folium sennae* extracts cause diarrhea by disrupting the function of tryptophan-metabolizing microbiota (Zhang et al., 2020).

Tryptophan can directly improve intestinal function by enhancing tight junction protein expression, as evidenced in an in vitro study (Liang et al., 2018b). Most of the effects of tryptophan are mediated by its metabolites. Tryptophan can be metabolized into many bioactive substances, including 5-HT, serotonin, and melatonin via the serotonin pathway, kynurenine via the kynurenine pathway, and indole and indole acid derivatives via intestinal commensal bacteria. Kynurenine, an agonist of the aryl hydrocarbon receptor (AhR), modulates intestinal immune capacity. Indole and indole acid derivatives modulate intestinal permeability and inflammatory responses. Additionally, several metabolites involved in this pathway are ligands for AhR and can promote barrier integrity, epithelial renewal, and activation of several immune cells by activating AhR signaling (Lamas et al., 2018; Sadik et al., 2020). 5-HT, as an antagonist of AhR, participates in the regulation of intestinal secretion, motility, and nutrient absorption (Modoux et al., 2021). 5-HT can be further metabolized to melatonin, which alleviates intestinal inflammation (Esteban-Zubero et al., 2017). Because of the crucial roles of tryptophan metabolism, impaired tryptophan metabolism is critically associated with various diet-related gastrointestinal diseases including diarrhea (Benech et al., 2021). Increased levels of tryptamine and 5-HT, products of tryptophan metabolized by host cells and gut microbes, respectively, have been observed in diarrhea-predominant irritable bowel syndrome (Agus et al., 2018; Mars et al., 2020).

Since tryptophan can be widely used by a variety of bacteria, dietary tryptophan has been shown to exert significant effects on gut microbiota composition and function in weaned piglets. Interestingly, different segments of the gastrointestinal tract respond differently to dietary tryptophan. For example, tryptophan decreased *Clostridium sensu stricto* and *Streptococcus* abundance and increased *Lactobacillus* and *Clostridium XI* abundance in the jejunum (Liang et al., 2018a), whereas it increased *Prevotella*, *Roseburia*, and *Succinivibrio* genera and decreased *C. sensu stricto* and *Clostridium XI* abundance in the cecum of weaned piglets (Liang et al., 2018b); Another study reported that dietary tryptophan reduced the abundance of *Prevotella,* and *Succinivibrio* genera, and enhanced *Ruminococcaceae* and *Lactobacillus* abundance in the colon of weaned piglets (Rao et al., 2021). These inconsistent results could be attributed to factors such as tryptophan concentration, dietary components, and animal physiological conditions. Nevertheless, these studies suggest that dietary tryptophan affects the gut function of weaned piglets via tryptophan-metabolizing bacteria.

Several studies have suggested that dietary tryptophan may exert side effects on intestinal function. Li et al. (2016b) reported reduced expression of tight junction proteins and enhanced intestinal permeability in piglets after tryptophan administration. Relatively high dietary tryptophan supplementation at a concentration of 0.75% negatively impacts jejunum morphology and tight junction function in weaned piglets (Tossou et al., 2016). Intestinal microbiota may use such a high concentration of tryptophan, resulting in an overaccumulation of 5-HT, which then activates the nerve response via the gut–brain axis to induce diarrhea (Spencer and Hu, 2020; Zhang et al., 2021). The double-edged effects of tryptophan may be determined by the dosage of amino acid, the nutritional ingredients of the diets and physiological conditions of the piglets.

### 3.4 Arginine

Oral supplementation with L-arginine and citrulline increases water and electrolyte secretion by enhancing NO production via activation of NO synthase in the small intestine (Grimble, 2007). NO is involved in the modulation of gut function, including maintaining water and electrolyte transport homeostasis and regulating motility throughout the intestine, which are both related to the occurrence of diarrhea. It has been suggested that low NO levels stimulate absorption, whereas high levels induce secretion. Thus, the dose of arginine is the main factor that determines its effects on the induction of diarrhea. A large single dose of poorly absorbed arginine may induce diarrhea, whereas low doses do not lead to side effects (Grimble, 2007). Additionally, large amounts of dietary amino acid supplementation can impair the hypertonic load by regulating gastric emptying, which further induces diarrhea. Arginine, a dibasic amino acid, is usually supplemented with a chlorine salt or salts of other anions, including aspartate or malate. These organic and chlorine anions exert synergistic effects, which can overwhelm the absorptive capacity of the intestine (Cynober et al., 1990). Thus, dipeptide forms of arginine are suggested to be better forms, since dipeptide absorption via di- and tri-peptide transporters (PEPT1) has higher efficiency (Wenzel et al., 2001). Furthermore, the effect of arginine is dependent on physiological and pathological conditions, especially when gastrointestinal motility and pharmacokinetics are affected.

Although arginine is widely used as a feed additive to improve intestinal health, especially in weaned piglets, few studies have reported its side effects, including diarrhea. In contrast, arginine supplementation in piglet diets increased feed intake and piglet growth, improved intestinal morphology and mucosa development, and alleviated oxidative and inflammatory responses due to its critical involvement in energy metabolism, functional amino acid synthesis, and cellular protein production, but not its role in NO synthesis. Several studies have suggested that dietary arginine decreases the diarrhea ratio (Che et al., 2019; Wang et al., 2012; Wu et al., 2010; Zhan et al., 2008) while others show no effect or do not provide results on diarrhea occurrence (Wu et al., 2012; Yao et al., 2011; Zheng et al., 2013, 2017). Most of these studies have shown that arginine improves villus height in the small intestine, indicating enhanced absorptive ability. This could be one reason dietary arginine alleviates diarrhea. The adverse effect on diarrhea incidence was only reported in a study which supplemented 1.6% arginine in piglet diets (Zheng et al., 2017), which is equal to the daily uptake of 9.6 g arginine based on 600 g daily feed intake. This is consistent with results of clinical trials which showed side effects when a single dose higher than 10 g arginine was given (Grimble, 2007). The dosage regimen used in piglet diets shows fewer adverse effects in many studies. It is possible that diarrhea could be ignored in those experiments since it might occur individually and infrequently in the late period of the experiment when the piglets have higher feed intake.

### 3.5 Sulfur-containing amino acids (SCAA)

SCAA are involved in critical cellular functions, as they participate in one-carbon metabolism. SCAA, especially cysteine, are rate-limiting substrates in the synthesis of glutathione, which is one of the main cellular antioxidants in the intestinal epithelium that mitigates weaning stress in piglets. As ROS play critical roles in inducing gut mucositis and diarrhea, the key role of SCAA in clearing free radicals indicates they likely confer beneficial effects against diarrhea. However, although SCAA exert many effects on gut health in weaned piglets, few studies have reported observation of direct effects of SCAA on the occurrence of diarrhea. One study reported that combined administration of cystine and theanine alleviated diarrhea (Yoneda et al., 2021). In addition to its major role in redox homeostasis, cysteine can also attenuate intestinal inflammation and improve mucosal barrier function and intestinal permeability in different models of inflammatory diseases (Kim et al., 2009; Song et al., 2016). Specifically, cysteine maintains intestinal immune homeostasis via promoting enhanced susceptibility of activated immune cells to apoptosis, and by enhancing nuclear translocation of NF-κB (p65) and Nrf2.

Methionine can also be used as a substrate for the synthesis of taurine and glutathione to neutralize oxidative stress and maintain gut homeostasis (Martinez et al., 2017). Several studies have demonstrated that dietary methionine helps maintain intestinal morphology, integrity, and barrier function in both normal post-weaned piglets and intrauterine growth-retarded piglets (Chen et al., 2014; Su et al., 2018; Zeitz et al., 2019; Zhang et al., 2019). Importantly, methionine decreases paracellular permeability by targeting tumor necrosis factor alpha (TNF-α) and alleviating inflammation (Martin-Venegas et al., 2013). Dietary supplementation with liquid DL-methionine hydroxy analog free acid in piglets increased the abundance of *Lactobacillus* spp. and decreased the abundance of *E*. *coli* in the rectum. These effects suggest that methionine may exert beneficial effects against diarrhea (Kaewtapee et al., 2016). Although the beneficial effects of SCAA have been widely reported, an imbalanced dietary methionine-to-sulfur amino acid ratio causes villous atrophy and exacerbates oxidative stress in weaned piglets (Bai et al., 2020). Thus, when extra methionine or cysteine is added to the diet, an adequate ratio of methionine to SCAA should be considered to maintain gut health and prevent diarrhea.

### 3.6 Glycine and serine

Glycine and serine are commonly considered nutritionally non-essential. However, recent studies have suggested that glycine and serine obtained by de novo synthesis are insufficient for piglet development, as they are metabolically necessary (Xu et al., 2022; Zhou et al., 2018b). Importantly, glycine is a major precursor of glutathione, and serine can be converted directly to glycine, catalyzed by serine hydroxymethyltransferase. Thus, these two amino acids are critical for modulating the antioxidant capacity of piglets.

Glycine exerts a wide range of beneficial effects on intestinal function, including improving antioxidant capacity, paracellular permeability, mucosal immunity, and energy status, and alleviating apoptosis. Dietary glycine exerts its effects in the intestines of weaned piglets by activating different signaling pathways. For example, Xu et al. (2018) found that glycine promoted protein synthesis via activating AMPK and mTOR pathways; Ji et al. (2022) and Xu et al. (2018) suggested that glycine alleviated inflammation via inhibiting Toll-like receptor 4 (TLR4), NF-κB and nucleotide-binding oligomerization domain (NOD) pathways; Yang et al. (2022) reported that glycine relieved apoptosis via the mTORC1 pathway. Although previous studies have confirmed that glycine enhances tight junction protein expression, the results have been inconsistent. Li et al. (2016a) found that physiological concentrations of glycine modulated expression and distribution of zonula occludens (ZO)-3 and claudin-7 proteins, but did not affect occludin, claudin-1, claudin-4, and ZO-2 in enterocytes isolated from the jejunum of newborn pigs. Fan et al. (2019) showed that maternal dietary glycine increased expression of occludin, ZO-1, and claudin-1 proteins, but did not affect claudin-3, ZO-2, and ZO-3 in weaned piglets.

Ferroptosis, a form of non-apoptotic, iron-dependent cell death that causes intestinal injury, is closely associated with intestinal oxidative stress. A recent study showed that, independently of its role as a substrate for glutathione synthesis, glycine can help maintain oxidative balance by targeting transferrin receptor protein 1 to eliminate ferroptosis (Xu et al., 2022). Since attenuated ferroptosis in the intestine is associated with a lower diarrhea score (Deng et al., 2021), glycine may decrease the occurrence of diarrhea by inhibiting ferroptosis. To date, direct evidence is lacking, and future studies exploring the mechanisms underlying the involvement of ferroptosis in diarrhea are warranted. Additionally, few reports mention the effects of glycine on gut microbes, except that dietary supplementation with 2% glycine decreased the abundance of pathogenic bacteria including Burkholderiales, *Clostridium* and *Escherichia-Shigella* (Ji et al., 2022). However, we did not find literature describing any relationship between these alterations and the occurrence of diarrhea.

Direct evidence shows that dietary serine improves growth performance and reduces the incidence of diarrhea (Zhou et al., 2018b). The effects of serine on gut health may be mainly attributable to its role in nucleotide and glutathione synthesis. Additionally, serine has been proven to alleviate oxidative stress by activating Nrf2 signaling, promote proliferation by activating mTOR signaling (He et al., 2020), and inhibit inflammation by eliminating NF-κB signaling in piglet intestines (Zhou et al., 2018b). However, studies on the application of serine to weaned piglets are limited. Recently, a dietary serine–microbiota interaction in which serine alters *E. coli*’s one-carbon metabolism was demonstrated (Ke et al., 2020). Thus, future studies on the mechanisms by which serine affects piglet diarrhea involving microbes are required.

### 3.7 Lysine

As the first limiting amino acid, lysine plays an indispensable role in protein synthesis and metabolic functions and is commonly sufficiently supplemented in the piglet diet. However, for those who are deficient in lysine, dietary lysine supplementation could reduce inflammation and diarrheal morbidity, independently of its physiological roles (Ghosh et al., 2010; Hayamizu et al., 2019). Studies using piglets as a model have found that lysine restriction causes apoptosis and affects the microbial composition in the intestine (Yin et al., 2017a, 2017b, 2018). Unfortunately, these studies did not record the incidence of diarrhea. Thus, whether these alterations in microbes are related to diarrhea remains to be explored.

### 3.8 Threonine

As one of the limiting amino acids in the piglet diet, threonine plays a critical role in intestinal development and piglet growth. Importantly, it has been suggested that 60% of dietary threonine is used for the synthesis of intestinal mucosal proteins including mucins (Mou et al., 2019), suggesting it provides beneficial effects on the maintenance of intestinal mucosal integrity and barrier function. Thus, a diet deficient in threonine exerts adverse effects in piglets. For example, a moderate threonine deficiency increases the paracellular permeability associated with villus hypotrophy and the expression of genes involved in defense responses (Hamard et al., 2007, 2010), indicating that threonine deficiency impairs intestinal integrity. Notably, neonatal piglets fed a threonine-deficient diet experienced lower acidic mucin levels accompanied with chronic diarrhea (Law et al., 2007).

Interestingly, dietary threonine deficiency and excess both decrease protein synthesis in the small intestine of pigs (Munasinghe et al., 2017; Wang et al., 2007). Specifically, piglets fed a threonine-deficient diet have reduced acidomucins and sulfomucins in the small intestine (Wang et al., 2010). The observation that limited threonine availability impairs gut protein synthesis, whereas an oversupply of threonine also causes the same outcome is reasonable. Dietary supplementation with threonine exacerbates colitis and extends the recovery period (Gaifem et al., 2018). Since threonine and neutral amino acids share the same transport system (Wu, 1998), it is possible that excess threonine competes with BCAA for transporters, resulting in reduced BCAA uptake. These results suggest that diets supplemented with balanced amino acids are important for protein metabolism and gut health.

### 3.9 Synergistic effects of different amino acids

Although most amino acids exert beneficial effects on gut health when used alone, a relatively high level of a single amino acid under certain conditions may cause side effects. Thus, amino acid mixtures are commonly used to supplement the diet to evaluate their synergistic effects. Combined supplementation with arginine and glutamine, rather than single amino acid supplementation, more strongly promoted villus development in the small intestine and reduced diarrhea incidence in weaned piglets (Shan et al., 2012). Diets supplemented with threonine and tryptophan together can be an alternative approach to antibiotics to prevent diarrhea (Engelsmann et al., 2023). Furthermore, low-dosage amino acid blends, including leucine, arginine, tryptophan, isoleucine, valine, and cystine, decrease the incidence of diarrhea in weaned piglets without affecting growth performance (Wessels et al., 2021). These studies suggest that combined supplementation with an amino acid mixture inhibits diarrhea better than amino acids used alone.

## 4 Conclusions and perspectives

Independently of their roles as substrates for protein synthesis, amino acids can exert a variety of beneficial effects on piglets, including improving intestinal integrity and permeability, and alleviating morphological damage and inflammatory and oxidative stress. Thus, appropriate supplementation could alleviate piglet diarrhea. Most of the effects of amino acids are mediated by metabolites, gut microbes, and related signaling pathways. Although many studies have evaluated the effects of dietary amino acids on gut health, most have not recorded the incidence of diarrhea. Therefore, future studies focusing on combined supplementation with functional amino acids, considering their balance in the piglet diet, are needed and their effects on preventing diarrhea should be specifically explored. Most of the studies in this review focused on the effect of amino acid supplementation on the incidence of diarrhea in weaned piglets. Because suckling piglets also exhibit a high incidence of diarrhea, future studies are encouraged to explore the effects of supplementing suckling piglets or lactating sows with extra amino acids.

## Author contributions

**Xihong Zhou**: writing - original draft. **Jing Liang**: writing - original draft. **Xia Xiong**: project administration, supervision, and writing - review & editing. **Yulong Yin**: project administration, supervision, and writing - review & editing.

## Declaration of competing interest

We declare that we have no financial and personal relationships with other people or organizations that can inappropriately influence our work, and there is no professional or other personal interest of any nature or kind in any product, service and/or company that could be construed as influencing the content of this paper.
